# Distributed Artificial Intelligence-as-a-Service (DAIaaS) for Smarter IoE and 6G Environments

**DOI:** 10.3390/s20205796

**Published:** 2020-10-13

**Authors:** Nourah Janbi, Iyad Katib, Aiiad Albeshri, Rashid Mehmood

**Affiliations:** 1 Department of Computer Science, Faculty of Computing and Information Technology, King Abdulaziz University, Jeddah 21589, Saudi Arabia; Njanbi0006@stu.kau.edu.sa (N.J.); IAKatib@kau.edu.sa (I.K.); AAAlbeshri@kau.edu.sa (A.A.); 2 High Performance Computing Center, King Abdulaziz University, Jeddah 21589, Saudi Arabia

**Keywords:** internet of everything (IoE), 6th generation (6G) networks, artificial intelligence, Distributed AI as a Service (DAIaaS), fog computing, edge computing, cloud computing, smart airport, smart districts

## Abstract

Artificial intelligence (AI) has taken us by storm, helping us to make decisions in everything we do, even in finding our “true love” and the “significant other”. While 5G promises us high-speed mobile internet, 6G pledges to support ubiquitous AI services through next-generation softwarization, heterogeneity, and configurability of networks. The work on 6G is in its infancy and requires the community to conceptualize and develop its design, implementation, deployment, and use cases. Towards this end, this paper proposes a framework for Distributed AI as a Service (DAIaaS) provisioning for Internet of Everything (IoE) and 6G environments. The AI service is “distributed” because the actual training and inference computations are divided into smaller, concurrent, computations suited to the level and capacity of resources available with cloud, fog, and edge layers. Multiple DAIaaS provisioning configurations for distributed training and inference are proposed to investigate the design choices and performance bottlenecks of DAIaaS. Specifically, we have developed three case studies (e.g., smart airport) with eight scenarios (e.g., federated learning) comprising nine applications and AI delivery models (smart surveillance, etc.) and 50 distinct sensor and software modules (e.g., object tracker). The evaluation of the case studies and the DAIaaS framework is reported in terms of end-to-end delay, network usage, energy consumption, and financial savings with recommendations to achieve higher performance. DAIaaS will facilitate standardization of distributed AI provisioning, allow developers to focus on the domain-specific details without worrying about distributed training and inference, and help systemize the mass-production of technologies for smarter environments.

## 1. Introduction

We are living in unprecedented times. Artificial intelligence (AI) has taken us by storm, helping us to make decisions in everything we do, even in finding the “true love” of our life and selecting the “significant other” [1]. Siri, Cortana, Google Assistant, Bixby, Alexa, Uber, Databot, Socratic, and Fyle are among the many apps that we use on an hourly basis, if not non-stop. The number of industries benefitting from AI is growing, such as recommender systems, autonomous vehicles, renewable energy, agriculture, healthcare, transportation, security, finance, smart cities and societies, and the list goes on [2,3,4]. The global market for AI is estimated to reach 126 billion U.S. dollars in 2025, from $10.1 billion in 2018 [5].

AI allows us to embed “smartness” in our environments by intelligently monitoring and acting on it [6]. Internet of Everything (IoE) extends the Internet of Things (IoT) paradigm and integrates various entities in this ecosystem including sensors, things, services, people, and data [7]. The grand challenge for such IoE enabled smart environments is related to the 4Vs of big data analytics [8]—volume, velocity, variety, and veracity—that is, to devise optimal strategies for migration and placement of data and analytics in these ubiquitous environments. The networking infrastructure would have to be smart to support these services and address the challenges.

**Figure 1:**
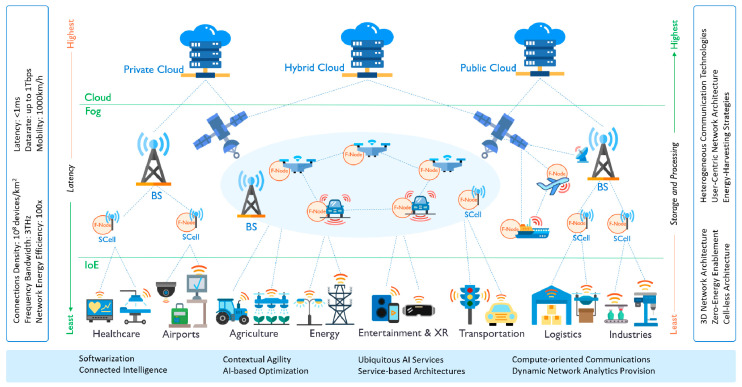
Sixth generation (6G)-internet of everything (IoE) enhanced smart societies.

Various deployments of the Fifth Generation (5G) of wireless systems have begun to appear across the globe, promising mobile internet at unseen speeds. However, a radical change is needed to support extreme-scale ubiquitous AI services [9,10,11,12]. the Sixth Generation networks (6G) pledge this through next-generation softwarization, heterogeneity, and configurability of networks [13,14]. 6G will provide much higher speeds, reliability, capacity, and efficiency at lower latencies [15] through various enabling technologies such as higher spectrum and satellite communications [9,10,16,17], the use of AI to optimize network operations, and the use of fog and edge computing [18,19]. Figure 1 (see Section 2 for elaboration) depicts an envisioned view of smart societies that are enhanced with 6G and IoE technologies, showing also the distinguishing characteristics of 6G.

The work on 6G is in its infancy and requires the community to conceptualize and develop its design, implementation, deployment, and use cases. Towards this end, this paper proposes a framework for Distributed AI as a Service (DAIaaS) provisioning for IoE and 6G environments. The AI service is “distributed” because the actual training and inference computations are divided into smaller, concurrent, computations suited to the large, medium, and smaller resources available with cloud, fog, and edge layers (see Figure 1). The AI service could be delivered by the Internet Service Providers (ISP) or other players. Multiple DAIaaS provisioning configurations for distributed training and inference are proposed to investigate the design choices and performance bottlenecks of DAIaaS. Specifically, we have developed three case studies (smart airport, smart district, and distributed AI delivery models) with eight scenarios (various usage configurations of cloud, fog, and edge layers) comprising nine applications and AI delivery models (smart surveillance, passport and passenger control, federated learning, etc.) and 50 distinct sensor and software modules (camera, ultrasonic sensor, electric power sensor, smart bins, data pre-processing, AI model building, data fusion, motion detector, object tracker, etc.).

The smart airport case study models the recently inaugurated King Abdulaziz International Airport, KAIA, Jeddah, and the smart district case study models the King Abdullah Economic City (KAEC), a smart city, both in Saudi Arabia. These two case studies model real-life physical environments and provide the details about actual sensors and computing operations and are used to understand and develop the DAIaaS framework and various AI service provisioning strategies. Using the knowledge gained from the first two case studies, the third case study investigates various distributed AI delivery models as a service (DAIaaS) without regard to the specific high-level applications in the underlying environments.

The evaluation of the DAIaaS framework using the three case studies is reported in terms of the end-to-end delay, network usage, energy consumption, and financial savings with recommendations to achieve higher performance. The results show a range of scenarios and configurations and how these affect the performance metrics and the related costs. Moreover, we demonstrate through these investigations and results that the challenging task for designing and deploying DAIaaS is the service placement because, for example, the edge devices might fail due to the limitations in their computation capabilities when the computing resource demands are high (as is the case for AI applications). Similarly, moving data too often to the cloud may lead to an inability to provision the required latencies for the edge devices.

The benefit of the DAIaaS framework is to standardize distributed AI provisioning at all the layers of digital infrastructure. It will allow application, sensor, and IoE developers to focus on the various domain-specific details, relieve them from worries related to the how-to of distributed training and inference and help systemize and mass-produce technologies for smarter environments. Moreover, to address the challenges noted by Viswanathan and Mogensen [19] and others, DAIaaS will provide unified interfaces that will facilitate the process of joint software development across different application domains. Therefore, we believe this work will have a far-reaching impact on developing next-generation digital infrastructure for smarter societies. To the best of our knowledge, this is the first work where distributed AI as a service has been proposed, modeled, and investigated.

The rest of this paper is organized as follows. Section 2 provides the background and reviews the related works. Section 3 explains our methodology and design, detailing how the various scenarios, applications, modules, and networks are modeled. Section 4, Section 5 and Section 6 give details that are specific to each of the three case studies and provide the performance analysis. Finally, conclusions are presented with future directions in Section 7.

## 2. Background and Related Works

We revisit Figure 1 that depicts a potential view of smart societies enhanced with 6G and IoE technologies. We consider the digital infrastructure of smart societies to be organized in three layers: IoE, Fog, and Cloud Layers. IoE Layer at the bottom comprises devices, sensors, and actuators from various application domains, transportation, energy, etc. The devices and sensors generate big data [20,21,22] that must be continuously processed and analyzed to make smart decisions and communicated to the IoE devices for actuation and other purposes. A sensor’s data may also be aggregated with other sensors for context-awareness, enhanced decision making, exploratory analyses, cross-sectoral and global optimizations, and other reasons (e.g., see [23]). The Fog Layer consists of fog nodes placed in various 6G connection providers (e.g., base stations) closer to the edge devices in the IoE Layer. Fog nodes will provide storage and computation power at the proximity of edges and with the 6G capabilities, they will achieve ultra-low latency. The Cloud Layer at the top consists of various types of private, public, and hybrid data centers (clouds) that will provide high computation and storage resources but with higher latency to the edges. Various distinguishing characteristics of 6G are mentioned in the boxes around the figure.

In the rest of the section, we explain and review the works related to the five core technologies used in our work. These are AI, IoE, edge-fog-cloud computing, smart societies, and 6G, discussed in Section 2.1, Section 2.2, Section 2.3, Section 2.4 and Section 2.5, respectively.

### 2.1. Artificial Intelligence (AI)

Artificial intelligence is a field of study that focuses on the creation of intelligent machines that can learn, work, and react intelligently like humans. Deep learning (DL), machine learning (ML), neural network (NN), pattern recognition, computer vision, natural language processing, clustering, etc. are tools that can be used to train computers to accomplish specific tasks such as computer vision and natural language processing (NLP) [23,24,25]. AI models usually rely on data to build their knowledge therefore big data and data collected from a huge number of devices and sensors, as in IoE, has provided the fuel for the AI models [4,20,21,22]. The increasing volume of data generated from many connected, heterogeneous, and distributed objects (IoT/IoE) and the continuous development and evolution of networks and communication technologies have motivated the emergence of Distributed Artificial Intelligence (DAI) [26]. In DAI, AI models are distributed into multi-agents (or multiple processes) that are cooperatively sharing knowledge to solve or act either separately or to build a global knowledge for the whole system. Agents or sub-models can be residing either inside a single machine or across multiple machines to perform AI training or inference in a distributed or parallel way. One approach is to partition the AI model into sub-models or sub-tasks that can be run concurrently using parallel processing techniques such as pipelining. Wang et al. [27] have developed a framework that pipelines the processing of partitioned NN layers across heterogeneous cores for faster inference. An alternative is data partitioning, where the dataset is split across concurrently-running models, and results are aggregated later. These techniques are useful with massive AI models and data. A discussion of model and data parallelism is available in [28,29].

Another approach is Edge Intelligence (EdgeAI) where the AI model is distributed across network edges. Several works have discussed the convergence of edge and AI [30,31,32,33,34,35]. AI model can be pre-trained then modified and optimized to be appropriate to run in the resource-constrained edges. A discussion of DL optimizations at both software and hardware levels for edge AI is covered in [36]. The collaboration between edge and cloud is also a possible model where some of the pre-processing and less-intensive computations are placed in the edge and global analysis located in the cloud. Parra et al. [37] have developed a distributed attack detection system for IoT where different AI models are used in both edge and cloud to provide local and global detection systems. Federated Learning (FL) is a DAI model where multi-agents collaboratively share their local knowledge for faster convergence and to make better decisions. FL concept, applications, challenges, and methods have been reviewed in [38,39]. Smith and Hollinger [40] developed a distributed robotic system that collaboratively shares their knowledge for a single goal (environment exploration). On the other hand, in [23] autonomous vehicles share knowledge to improve their own decisions.

Artificial Intelligence-as-a-Service (AIaaS) has also been considered in [41,42,43] as a natural extension of the usual “as-a-service” (“aaS”) service delivery models available with cloud providers. This allows developers to focus on their domain-specific details and conveniently add AI capability to their software. Several works have shown the benefits of AIaaS in developing and supporting applications that require AI capabilities. Casati et al. [42] proposed a framework and architecture that facilitate the deployment of cloud-based AIaaS for smarter enterprise management solutions. Milton et al. [43] conducted real experiments utilizing Google’s Dialogflow API (a simple AIaaS provided by Google) [44] to develop a chatbot. The AI services that are provided by the top cloud providers such as Google, Amazon, Microsoft, and IBM, are discussed in [41].

### 2.2. Internet of Everything (IoE)

Internet of Everything (IoE) has emerged as a concept that extends the Internet of Things (IoT) [45] to include processes, people, data, and things [7]. In the core of IoE, sensors are usually embedded with “everything” to monitor, identify the status and act intelligently to generate new opportunities for the society. There are a variety of sensors designed for different purposes such as temperature, pressure, biosensors, light, position, velocity, etc. which are discussed in [46]. A massive number of sensors are expected to be deployed everywhere to support such applications and many others in different areas including industrial, traffic, smart cities, and healthcare systems [47,48].

There are some IoE works that have looked at challenges in sensors connection and data collection and processing. AlSuwaidan [49] adopted Cloud as a Service (CaaS) and the Fog-to-Cloud concept to overcome the challenge of integrating, storing, and migrating distributed data. Lv and Kumar [50] proposed the software definition to sensors in the 6G/IoE network, along with Software Defined Network (SDN) technology to provide better control. Aiello et al. [51] have developed a self-contextualizing service for IoE that separates the logical part from the physical contexts. Others such as Badr et al. [52] focused on energy harvesting and Ryoo et al. [53] covered security and privacy concerns in IoE.

### 2.3. Edge, Fog, and Cloud Computing

The continuous increase in the number of IoE sensors and edge devices joining the network required a shift in the paradigm that pushes data processing closer to the data sources. Edge and fog computing are two architectures that aim to bring processing closer to users at the network edges. While some in the literature do not differentiate between fog and edge [54], we and many others differentiate between them [55,56], depending on where the computation is performed. In edge computing, processes are localized in the edge devices to produce instant results. On the other hand, fog computing is an intermediate layer extending the cloud layer that brings the functions of cloud computing closer to the users [57]. Fog nodes are devices that can provide resources for services, and they might be resource-limited devices such as access points, routers, switches, and base stations, or resource-rich machines such as Cloudlet and IOx [58].

Discussions on fog computing and other edge paradigms are provided in [50,59] and their role in IoT is covered in [60]. Nath et al. [61] proposed an optimization algorithm to manage communication between the IoE cluster and the cloud. Wang et al. [62] adopted imitation learning for online task scheduling in vehicular edge computing. Badii at al. [63] have developed a platform for managing smart mobility and transport in network edges. Tammemäe et al. [64] proposed service architecture to support the self-awareness in fog and IoE.

### 2.4. Smart Cities, Societies, and Ecosystems

Smart cities and smart ecosystems employ different information and communication technologies (ICT) to intelligently monitor, collect, analyze, respond to environmental changes [2,65,66,67,68,69,70,71,72,73,74,75]. The population growth in the urban area and the advancement in technologies have increased the demand for more sustainable cities that adopt smarter, effective, and efficient ways to manage the urban area and integrate various aspects of the ecosystem [76]. This includes introducing smartness to the infrastructure, operations, services, industries, education, security, and many more. In this context, IoE will be the base that will enable and integrate city services, people, things, and data. Deploying sensors all over the city including the one that is attached to the people, such as smartwatches, or at their mobile devices will provide great services and unlimited innovation opportunities.

Several works have looked at the design of the applications for smart societies. Ahad et al. [77] have developed a smart educational environment based on IoE to produce a learning analytics system that evaluates the learning process and achievements. Al-dhubhani et al. [78] have proposed a smart border security system where sensors and different sources of data are used to make decisions and take actions. Queralta et al. [79] proposed an IoE-based architecture that employs a heterogeneous group of vehicles to improve traveling quality. Alam et al. [80] have developed an object recognition method for autonomous driving to improve the accuracy of vehicle recognition. Many other proposals on smart societies [81] exist, such as in transportation [25,65,69,71,82], healthcare [6], disaster management [83], logistics [66,84], and more.

### 2.5. Sixth Generation Networks (6G)

6G is the next generation of cellular networks that is expected to overcome the limitation of current fifth-generation (5G) deployment and fulfill requirements of the future fully connected digital society [10]. There are some publications [10,13,14,15,16,17,18,19] that have discussed the future vision of 6G cellular networks, requirements, enabling technologies, and challenges. The main challenges are coming from the expected continuous increase in the number of sensors joining the network and the popularity of IoE-based smart services [9]. Extensive improvements in the speed and capacity of communication can be achieved by adopting a higher spectrum and utilize various communication technologies [15]. 6G networks are expected to be an ultra-dense heterogeneous network [85]. Both terrestrial (cellular network) and non-terrestrial (e.g., satellites, drones, and planes) infrastructures will be employed to provide continuous and reliable network services [10]. Though the heterogeneity of network architecture, communication links, devices, and applications will increase network control and operation complexity [17]. Therefore, 6G is expected to take the 5G softwarization and virtualization to their next level by empowering the network with AI approaches to optimize the network operation [13,14]. Moreover, service-oriented operations should offer higher flexibility and looser integration with various network components, which will facilitate deployment, configuration, and management of new applications and services [16]. Distributed artificial intelligence with user-centric network architectures will be a fundamental component of the 6G networks to reduce communication overhead, and provide autonomous and real-time decisions [10]. Energy efficiency is also one of the critical requirements for 6G networks that must be taken into account from antenna design to the zero-energy nodes for low rate sensing applications [9]. It is expected from 6G to have 10–100 times higher energy efficiency than 5G to accommodate joining devices and applications with lowest-cost and eco-friendly deployment [11].

To summarize, digital infrastructures that will support smart societies require rich and flexible AI capabilities. 6G pledges to support ubiquitous AI services, however, the work on 6G is in its infancy and requires the community to contribute to its realization, such as in designing AI models, data management, service placements, job scheduling, and communication management and optimization for both application developers and service providers. Solutions are required to reduce the complexity of the systems and to allow application developers to focus on the various domain-specific details rather than worrying about the how-to of distributed training and inference. Table 1 summarizes some of the reviewed literature and compares it with our work. Note in the table that none of the published proposals have incorporated all the key technologies for next-generation digital infrastructure. The particular differentiating factor of our work is the DAIaaS framework and its detailed evaluation. 

## 3. Methodology and Design

In Section 1, we have already given an overview of our methodology in terms of the motivation for the three case studies, and the comprising scenarios, applications and AI delivery models, and sensor and software modules. This section discusses our methodology and design, and the main components of our simulations in detail. Section 3.1 describes the devices used in the edge, fog, and cloud layers. Section 3.2 introduces the applications and delivery models used in our work and how these are modeled in the simulations. Section 3.3 explains how the network infrastructure is modeled. Finally, Section 3.4 defines the performance metrics used for performance evaluation.

Software and Hardware: We have used the iFogSim [86] simulation software to model and evaluate DAIaaS. We selected it because it allows simulating a range of applications, modules, placements, data streams, sensors, edges, fogs, cloud datacenters, and communication links. All experiments are executed on the Aziz supercomputer (Jeddah, Saudi Arabia), which comprises 492 nodes with 24 cores each. The supercomputer allowed us to run many large simulations with different configurations concurrently on different nodes.

### 3.1. Cloud, Fog, and IoE Layers

Figure 1 shows a high-level view of smarter environments, supported by 6G and IoE, comprising three main layers: IoE, Fog, and Cloud, and this has been explained in some detail in Section 2. Each layer contains devices with distinct resource capabilities that are represented in the simulations using various parameters. We have determined the values for these parameters considering the specification of the devices that are available today. Table 2 list the three types of devices (edge, fog, and cloud devices) and the associated parameters. The computational capabilities of these devices are represented in the simulations with certain values of MIPS (Million Instructions Per Second) and RAM (Random Access Memory). The communication capabilities are simulated using uplink and downlink bandwidth. Each device is also characterized by specific power consumption in the idle and busy states. For example, The MIPS parameter for cloud for one virtual machine (VM) has the highest MIPS and RAM (220,000 and 40,000) values compared to the fog and edge devices.

### 3.2. Distributed Applications and AI Delivery Models

**Figure 2:**
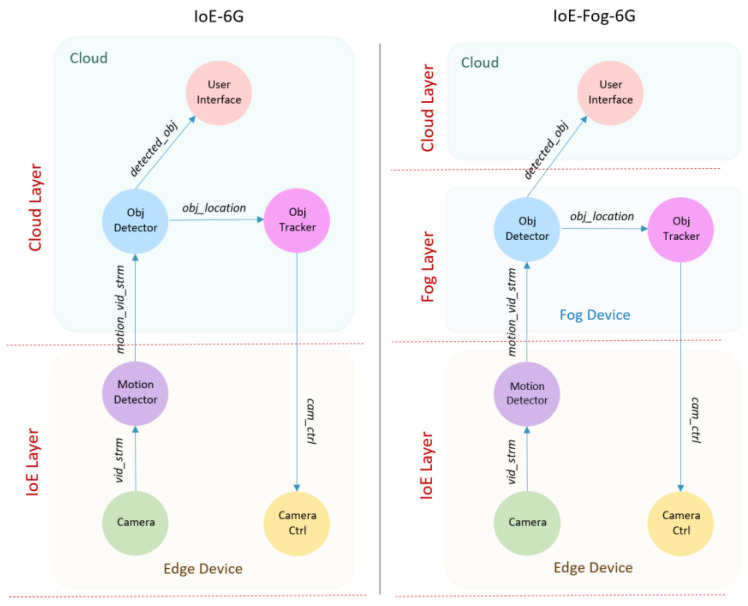
Smart surveillance application module.

A smart city would have various applications running on it simultaneously. Each application (see Section 4 and Section 5) and AI delivery models (see Section 6) has a set of modules (*m*) that performs some computations on the data they receive. The modules are organized in a directed graph (DG) with edges between them to represent data or workload (*w*) passing between the modules. This is depicted in Figure 2 using the Smart Surveillance application, which we use in this section as an example. For each workload received by the module, it will be processed and a new workload will be generated depending on the configured mapping between workloads and the selectivity rate in case more than one exists. A workload (*w*) can be characterized by its CPU requirements (*w_c_*) in terms of million instructions (MI) required by the module to process the workload as well as its network requirements (*w_n_*) in terms of bytes to be transferred between the two modules over the network.

Table 3 lists the various workloads used in the Smart Surveillance application. We will come back to it later after explaining the application in Figure 2. The figure shows that the modules can be placed in different layers (i.e., edge, fog, or cloud devices) depending on the scenario (IoE-6G versus IoE-Fog-6G). The Smart Surveillance application uses CCTV (Closed-Circuit Television) cameras to detect and track objects in a specific area, such as in [86]. The CCTV cameras generate live video streams. Therefore, this application has a high computation requirement especially in a crowded environment such as airports or pedestrian areas, where many people and objects must be tracked, identified, and analyzed carefully for security reasons. The application consists of six modules: Camera, Motion Detector, Object Detector (Obj Detector), Object Tracker (Obj Tracker), Camera Control (Camera Ctrl), and User Interface.

The Camera contains the sensor and Camera Ctrl contains the pan–tilt–zoom (PTZ), which is the actuator in the camera that adjusts the camera zoom depending on the PTZ parameters. The Motion Detector is always located in the smart cameras and it receives live video streams (*vid_strm*) from the Camera and when motion is detected it transfers the motion video stream (*motion_vid_strm*) to the Obj Detector module. The Obj Detector module is located in the cloud in the IoE-6G scenario, and in the fog node in the IoE-Fog-6G scenario. It receives video streams (*motion_vid_strm*) from the Motion Detector and intelligently detects objects and activates Obj Tracker if it hasn’t been activated before for the same object. The Obj Detector module sends two workloads: the detected object (*detected_obj*) to the User Interface and the object location (*obj_location*) to the Obj Tracker. The Obj Tracker module is located in the cloud in the IoE-6G scenario, and in the fog node in the IoE-Fog-6G scenario. It receives coordinates of the tracked objects (*obj_location*) and calculates the PTZ configuration, which is sent to the Camera Ctrl using the workload, camera control (*cam_ctrl*). The User Interface is always located in the cloud and it receives a video stream of the tracked objects (*detected_obj*) from the Obj Detector. Each application contains one or more *application loop*, which is defined as a series of modules (a tuple) to measure the end-to-end delay between the start and the end of the loop. The Smart Surveillance application contains one control loop represented by the tuple of modules (Camera, Motion Detector, Obj Detector, Obj Tracker, Camera Ctrl). The end-to-end delay of each *application loop* defined in Section 3.4 and is computed as part of the application and network performance.

Table 3 lists the configuration of each workload, specified with its source and destination modules and resource requirements. For example, Row 1 in the table shows that the workload *vid_strm* requires 1000 million instructions (MI) and 20000 bytes to be transferred from the Camera module to the Motion Detector module.

### 3.3. Network Infrastructure

We have defined five categories of devices, Cloud, Gateway, Fog, Edge, and Sensor/Actuator. These devices operate in different layers and accordingly the expected link latency between them varies. Table 4 lists the various types of links (*L*) and their defined latency (*t_l_*) in ms. These latencies are set based on the expected 6G link latencies between the layers. For instance, the configured link latency between Cloud and Gateway is set to 100 ms while links between Gateway, Fog, and Edge are set to 2 ms because they are closer to each other. The link latency between Edges and their Sensor/Actuator is set to 1ms because they are expected to be part of the edge devices. We have deliberately used modest values for latencies compared to the expected 6G latencies to keep some levels of performance margins.

### 3.4. Performance Metrics

For evaluation purposes, three performance metrics are monitored: network usage, application loops end-to-end delay, and network energy consumption.

The network usage (*U*) is the average load on the network in bytes per second. *U* is computed by Equation (1) where *t_l_* represents the latency of a link *l* and *t_L_* is a set of all links latency. *W_n_* is the network requirement of workload *w* and *W_n_* is a set of all workload’s network requirements. *T* is the total simulation time.

(1) $$Network\;usage\;U=\frac{\sum_{t_{l}\in t_{L},w_{n}\in W_{n}}\;w_{n}*t_{l}}{T}$$

The application’s loop end-to-end delay allows us to evaluate the response time of the applications in different scenarios. For every application loop type (*a*), we calculate the average end-to-end delay (*D_a_*) from the first module to the last module in a specific loop using Equation (2). *T_s(i)_* is the start time and *t_e(i)_* is the end time of loop number (*i*) of type *a*, and *I* is the total number of loops of type *a*.

(2) $$Loop\;delay\;D_{a\in A}=\frac{\sum t_{e\left(i\right)}-t_{s\left(i\right)}}{I}\;,\;0<i<I$$

The network energy consumption is calculated per hour (*E_h_*) using Equation (3) where *ε* is the estimated energy and *U* is the network usage. To estimate the network energy consumption (*E_h_*), we used the energy estimation of a gigabyte transfer on the network from [87] Table 5 shows their forecasted energy consumption rate for the wireless access network (WAN) for 2010, 2020, and 2030. The average energy consumption of 2020 = 0.54 kWh/GB used as *ε* value.

(3) $$Energy\;consumption\;E_{h}=\;3600*\varepsilon *U$$

In addition to the network energy consumption, the estimated daily Cost of energy is also calculated based on the electricity price in Dollar per kWh for Saudi Arabia from [88] using Equation (4). *β* is the electricity price in dollar per kWh and *E_h_* is the energy consumption per hour.

(4) $$Cost\;C_{d}=\;24*\beta *E_{h}$$

## 4. Case Study 1: Smart Airport

In this section, we present and discuss our first case study (Smart Airport) including the use of IoE in smart airports and their applications, the experiment design, configuration, and results.

### 4.1. IoE in Smart Airports

According to the International Air Transport Association (IATA), it predicted that the number of passengers will double to 8.2 billion by 2037 [89]. This expected increase in the number of passengers will put huge pressure on the aviation industry, especially in the current infrastructure [89]. IoE will also play an important role in enhancing passenger experience and offering a great opportunity for both airlines and airports [90]. Many devices and sensors can be deployed to support the smartness in the airport such as surveillance cameras, Radio-frequency identification (RFID), various sensors (e.g., air quality sensor), wearable devices (e.g., watches), avionics devices (e.g., flight recorders), biometric devices and/or, digital regulators (e.g., electricity). Using data collected from these devices, many smart airport applications might be adopted such as Baggage Tracking, security applications, indoor navigation systems, and airport operation and administrations.

### 4.2. Smart Airport: Architectural Overview

**Figure 3:**
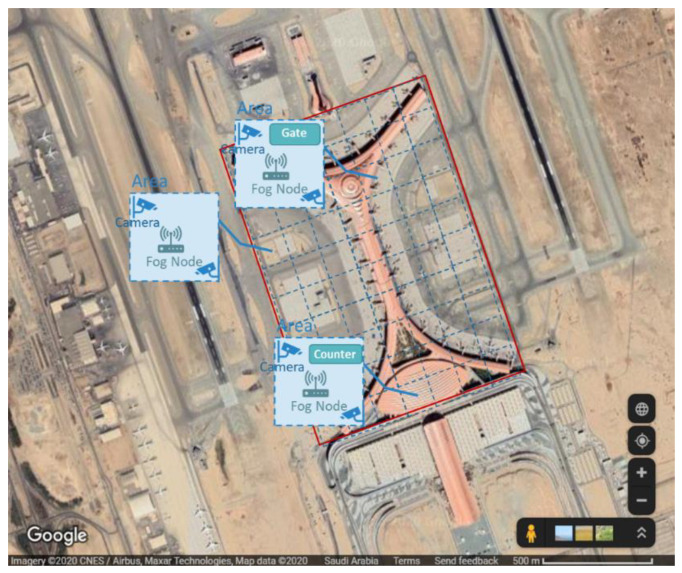
King AbdulAziz International Airport (Smart Airport Layout).

**Figure 4:**
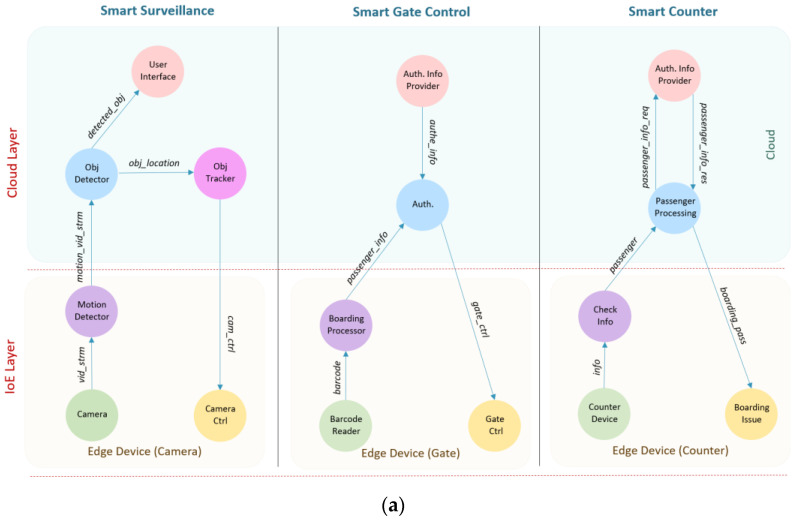

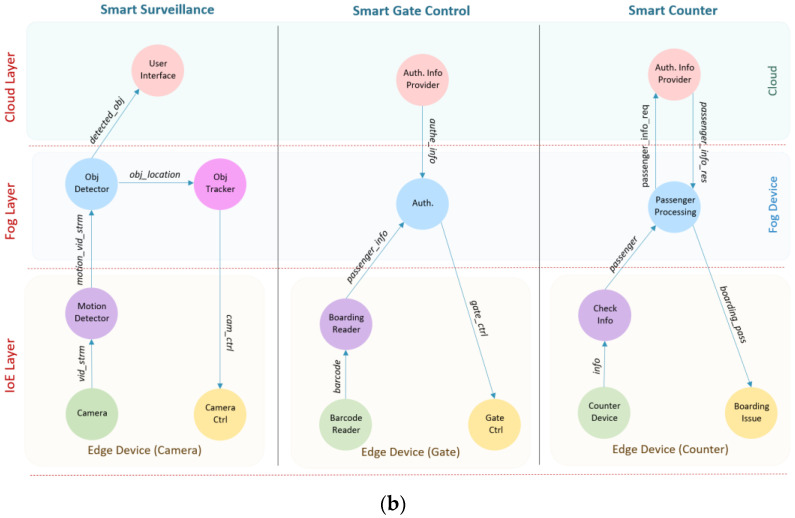
Smart airport: (**a**) IoE-6G scenario and (**b**) IoE-Fog-6G Ssenario.

In the first case study, we selected the new King Abdulaziz International Airport (KAIA), Jeddah, Saudi Arabia, for evaluation. Figure 3 shows the layout of the simulated airport including the main components of the system. The whole airport landscape is divided into small areas, where each area is covered by a gateway router that works as a fog device. This router provides a connection for all edge devices in that area. Three types of edge devices are simulated: smart camera, barcode readers (at gates), counter devices, and each of them is connected to a specific type of sensor or actuator. Figure 4 shows the detailed architectural design of the IoE-6G (a) and IoE-Fog-6G (b) scenarios. Three applications are shown Smart Surveillance, Smart Gate Control, and Smart Counter. Although both scenarios have the same physical infrastructures, the application modules placement differ in them. In the following sections, we will discuss Smart Gate Control and Smart Counter, while third application, Smart Surveillance, we explained already in the previous section.

### 4.3. Application: Smart Counter

The Smart Counter application is responsible for counter operation where passengers finish their check-in procedures. The application consists of five modules: Barcode Reader, Check Information (Check Info), Passenger Processing, Authentication Information Provider (Auth. Info Provider), and Boarding Issue. The Barcode Reader uses light sensors to read passports or ID cards. The Check Info module receives passenger information (*info*) from the counter and passes it to the Passenger Processing module. Passenger Processing is located in the cloud at the IoE-6G scenario, and in fog at the IoE-Fog-6G scenario. It receives passenger (*passenger*) orders from the counter and requests passenger information (*passenger_info_req*) from the Auth. Info Provider to perform the check-in process. The Auth. Info Provider will send the result back (*passenger_info_res*) to Passenger Processing module. After authentication, the boarding pass information (*boarding_pass*) will be sent to the Boarding Issue actuator. Auth. Info Provider is always located in the cloud. In the case of the IoE-Fog-6G scenario, it has an extra role, that is designed to increase the data locality and provide faster service at the smart gates. When a passenger arrives at the counter and after the check-in, the passenger authentication information (*authe_info*) will be sent to the fog node where the passenger boarding gate is located. In this way, when the passenger arrives at the gate, his information will be available at the fog which will enhance the response time of the gate. Auth. Info Provider will send periodically the passenger’s data to the proper fog node, specifically to the Authenticator (Auth.) module of the Gate Control application, which will be discussed next.

### 4.4. Application: Smart Gate Control

The Smart Gate Control application is responsible for processing passengers boarding passes at boarding gates. The application consists of five modules: Counter Device, Boarding Processor, Authenticator (Auth.), Authentication Information Provider (Auth. Info Provider), and Gate Control (Gate Ctrl). The Barcode Reader uses light sensors to read boarding pass code. The Boarding Processor module is always located in the smart gate. It receives barcode information (*barcode)* from the Barcode Reader, and it sends the code to the Auth. for authentication. The Auth. is located in the cloud on the IoE-6G scenario, and fog node on the IoE-Fog-6G scenario. It receives passenger info (*passenger_info)* from the Boarding Processor and authenticates the passenger. After that, a decision will be sent as a control signal (*gate_ctrl*) to the Gate Ctrl, so it acts depending on that. The Auth. Info Provider is placed in the cloud and is responsible for communication with the Auth. Info Provider in the Smart Counter application.

### 4.5. Experiment Configurations

The main configuration parameters of the airport simulation are the number of areas, cameras, gates, and counters. As mentioned in the previous section, the whole airport is divided into areas, each with a fog device and a set of cameras, gates, and counters. KAIA main building area is around 1.2 km^2^, therefore we assumed that each simulated area is around 100 m^2^ and ranged our areas parameter from 100 to 1000 to cover the 1 km^2^, having 10 configurations in total. Each area has two cameras (ranging from 200 to 2000 cameras), and in total 40 gates and 120 counters distributed across different areas. These configurations aim to show the architecture performance when the whole environment scales up from 360 to 2160 devices.

Table 6 lists the configurations of the Smart Airport sensors including the type of workload they generate, and the distribution of inter-arrival time. Camera has deterministic distribution as it generates workloads regularly every 5 ms while gate and counters have uniform distribution (5 to 20 ms) as they depend on the passenger arrival. Table 7 lists the various workloads alternating between modules for the Smart Gate Control and Smart Counter applications, while the Smart Surveillance workloads is listed in Table 3.

### 4.6. Results and Analysis

**Figure 5:**
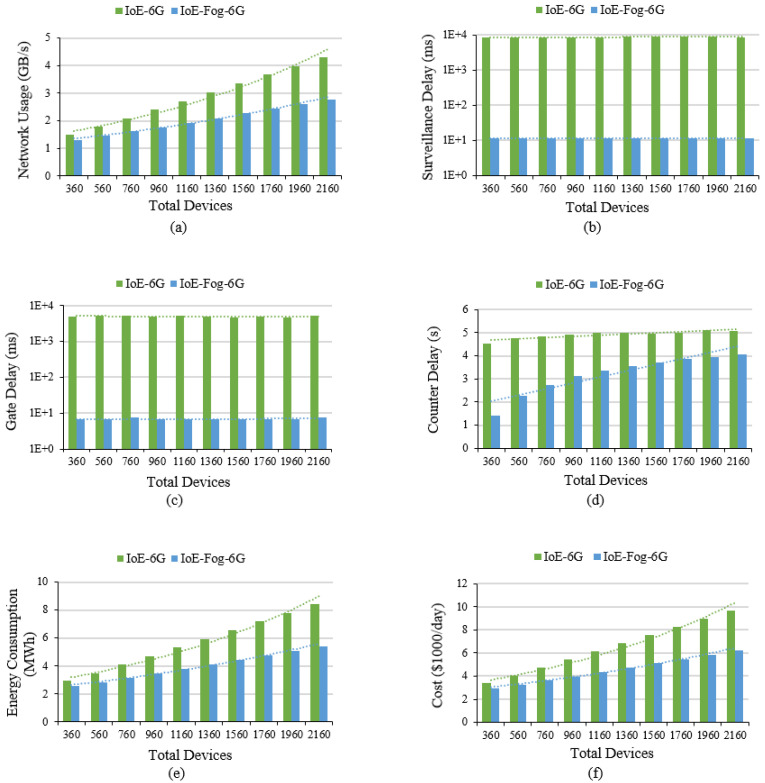
Smart airport case study results: (**a**) Total network usage, (**b**) Smart surveillance application average loop end-to-end delay on a log scale, (**c**) Smart gate application average loop end-to-end Delay on a log scale, (**d**) Smart counter application average loop end-to-end delay, (**e**) Network energy consumption, and (**f**) Estimated energy cost.

This section will present our results of IoE-6G and IoE-Fog-6G scenarios for the 10 configurations in terms of network usage, application loop end-to-end delay, and energy consumption. Figure 5a shows the network usage in GB/s. Deploying modules on fog devices in the IoE-Fog-6G scenario decrease the volume of data sent to the cloud by 36% for 2160 devices. The difference between the two scenarios network usage increased from 13% to 36% with the increase in the number of devices which approves that IoE-Fog-6G architecture will have a greater impact when the network scale-up and will alleviate traffic jams around the datacenter.

Figure 5b shows the average application loop end-to-end delay of the two scenarios for the Smart Surveillance control loop. The main control loop for Smart Surveillance is the tuple of modules (Camera, Motion Detector, Obj Detector, Obj Tracker, Camera Ctrl). There is a huge difference in the delay of the applications between the two scenarios. The IoE-Fog-6G provided a faster response than the IoE-6G as most of the processing is done on the edge and fog devices. In addition, because the data here is a stream of video, a huge amount of time is reduced when the unnecessary transformation is avoided. In Figure 5c, the average application loop end-to-end delay for Smart Gate is shown. The tuple (Barcode Reader, Boarding Processor, Auth., Gate Ctrl) is the main control loop for the Smart Gate. Similar to Surveillance, the delay is significantly less in the IoE-Fog-6G scenario. In addition, here we can see the benefit of proactive caching that the Smart Counter performs when it processes a passenger as the passenger authentication information is transformed into the gate fog. This guarantees the availability of authentication information before the passenger arrival which improved the loop latency. Finally, the average application loop end-to-end delay for a Smart Counter is shown Figure 5d. The tuple (Counter Device, Check Info, Passenger Processing, Auth. Info Provider, Passenger Processing, Counter Ctrl) is the main control loop for the Smart Counter application. The result here differs from the results of Surveillance and Gate applications because of the way modules are placed in this application. As shown in Figure 4, the Auth. Info Provider module is always located in the cloud in both scenarios, so when the number of devices increases the pressure on the datacenter increase which means higher delay.

Figure 5e shows the average energy consumption for network data transfer. The energy consumption is reduced in the IoE-Fog-6G scenario by around 3 MWh with 2160 devices, and this difference is also expected to be larger with more devices joining. Similarly, Figure 5f shows the cost of energy for 2160 devices reduced by $3500 per day with fog deployment which is around $1,260,000 per year saving.

## 5. Case Study 2: Smart District

In this section, the second case study is presented which evaluates the effectiveness of deploying edge/fog layer to the IoE/6G network in a smart district system. This case study differs from the smart airport case study as it represents an outdoor area and uses the 6G base stations as fog devices. In the following subsections, we will discuss the use of IoE in the smart district and its applications. Then, our experiment design, configuration, and results will be presented.

### 5.1. IoE in Smart District

The smart district is the building block for smart cities and various applications can be involved to provide the smartness that will improve the quality of life. Challenges such as energy and utility provision, healthcare, education, transport, waste management, environment, and many others must be solved efficiently and effectively in a smart ecosystem [91]. Smart sensors and IoE devices provide real-time monitoring of the district and act inelegantly. Applications such as parking guidance systems, parking lots monitoring, parking lot reservation, parking entrance, and security management, use sensors such as infrared sensors, ultrasonic sensors, inductive loop detectors, cameras, RFID, magnetometer, and/or microwave radar [92]. For energy provisioning and management systems, smart grids might be utilized to provide a real-time monitoring and control using sensors that can read parameters such as voltage, current, power flow, and temperature [93].

### 5.2. Smart District: Architectural Overview

**Figure 6:**
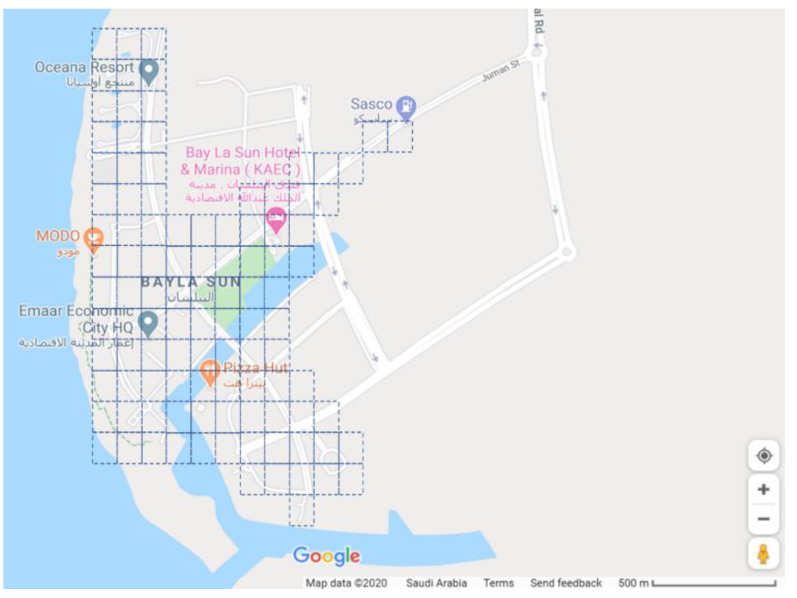
Simulated King Abdullah Economic City’s (KAEC’s) Bayla Sun District Layout.

**Figure 7:**
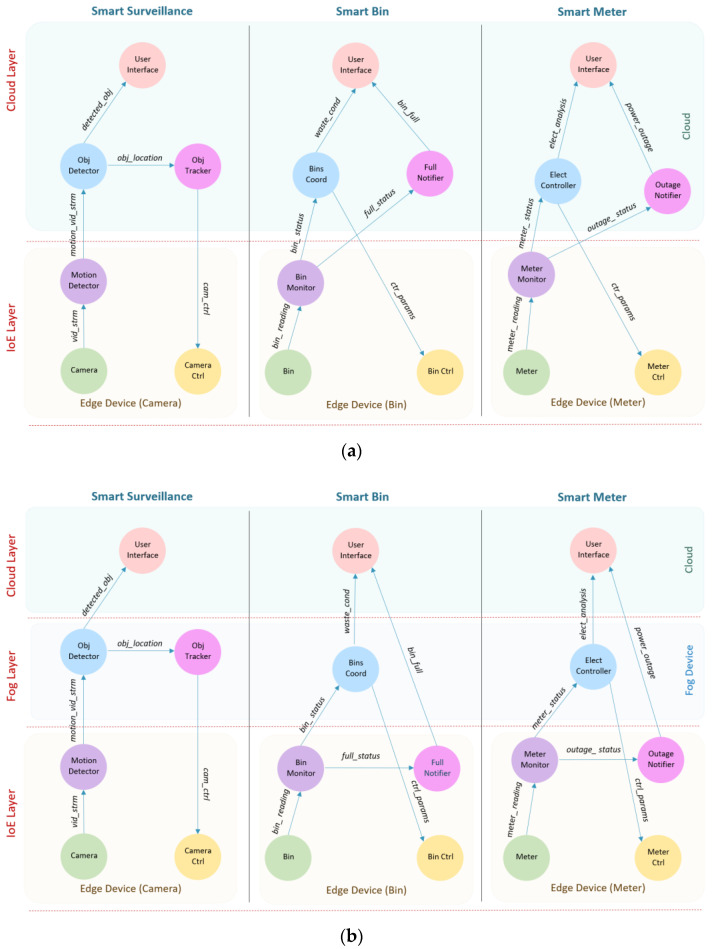
Smart District: (**a**) IoE-6G Scenario and (**b**) IoE-Fog-6G Scenario.

King Abdullah Economic City (KAEC) is one of the new cities in Saudi Arabia that aims to provide a new way of living, working, and playing. KAEC is around 173 km^2^, so we choose one of its districts that is the Bayla Sun district which is around 4 km^2^. Bayla Sun is one of the active areas at KAEC and has different facilities including, a residential area, college, parks, hotels, and a resort, a fire station, restaurants, and many others. Figure 6 shows a screenshot of Bayla Sun district from google map. The active area is divided into small areas of a size around 100 m^2^. Each is supported by one fog device (6G station). Three applications of the smart district are simulated Smart Surveillance, Smart Meters, and Smart Bins. Three types of edge devices: Smart Camera, Smart Meter, and Smart Bin are considered, and each of them is connected to its specific sensor or actuator depending on the system. The IoE-6G or IoE-Fog-6G scenarios have the same physical infrastructures but different modules placemen. Figure 7 shows the detailed architectural design of both scenarios and their application modules placement. In the following subsections, we will discuss the Smart Meter and Smart Bin applications. Smart Surveillance application were already explained in the previous sections.

### 5.3. Application: Smart Meter

The Smart Meter application is the energy management and analysis system in the district where voltage and current sensors monitor electricity usage and detect power cut in a real-time manner. This application consists of six modules: Meter, Meter Monitor, Electricity Controller (Elect Controller), Outage Notifier, User Interface, and Meter Control (Meter Ctrl). The Meter Monitor module is always located in the smart meter device. It receives reading (*meter_ reading*) from the Meter sensors and it sends the reading to the Elect Controller. It also detects an outage and sends an outage signal (*outage_ status*) to the Outage Notifier. The Electricity Controller module is the main processing module and it is located in the cloud on the IoE-6G scenario and fog node on the IoE-Fog-6G scenario. It receives status (*meter_ status*) from Meter Monitor to be evaluated and will send electricity analysis (*elect_analysis*) to the User Interface and controls (*ctrl_params*) to Meter Ctrl. The Outage Notifier module is also located in the cloud on the IoE-6G scenario and in meter (edge) on the IoE-Fog-6G scenario and it receives an outage signal (*outage_ status*) from the Meter Monitor and sends an outage signal to the local operator. The User Interface is always located in the cloud and it receives electricity analysis (*elect_analysis*) and presented to the user.

### 5.4. Application: Smart Bin

The Smart Bin application is the smart waste management system that optimize and monitor waste collection and recycling in a real-time manner. Smart Bins have sensors that use ultrasonic beams to sense fill-levels and type of waste, such as mixed waste, paper, glass, or metal. This application consists of six modules: Bin, Bin Monitor, Bins Coordinator (Bins Coord), Full Notifier, User Interface, and Bin Control (Bin Ctrl). The Bin Monitor module is always located in the smart bin device. It reads the bin fill-levels from the sensors and it sends the reading (*bin_ reading*) to the Bins Coord. It also detects a full bins state and sends a full signal (*full_status*) to the Full Notifier for real-time responses. The Bins Coord module is the main waste management module and it is located in the cloud on the IoE-6G scenario and fog node on the IoE-Fog-6G scenario. It receives bin status (*bin_ status*) from Bin Monitor to be analyzed and will send waste conditions (*waste_cond*) to the User Interface and controls (*ctrl_params*) to Bin Ctrl. The Full Notifier module is located in the cloud on the IoE-6G scenario and bin on the IoE-Fog-6G scenario. It receives a full signal (*full_status*) from the Bin Monitor and sends a full signal to the local operator for collection. The User interface module similar to other applications is always located in the cloud. It receives waste conditions (*waste_cond*) from the Bin Monitor and presents it to the user.

### 5.5. Experiment Configurations

The main configuration parameters of the district simulation are the number of areas, cameras, meters, and bins. The number of areas is fixed to 100 to represent the active areas of KAEC’s Bayla Sun district as shown in Figure 6. In each area, we specify the number of cameras, meters, and bins on it. In this study, 10 configurations were also simulated. The aim here is to show the architecture performance when the granularity of IoE devices increases. Therefore, the number of cameras, meters, and bins is increased from 1 to 10 per area (fog device) which means that the total number of end devices ranges from 300 to 2100 devices. The sensors in this case study are configured to periodically generate workloads following a deterministic distribution of 5 ms. Table 8 list the properties of all workloads alternating between application modules for the two applications, while the Smart Surveillance is listed in Table 3.

### 5.6. Results and Analysis

**Figure 8:**
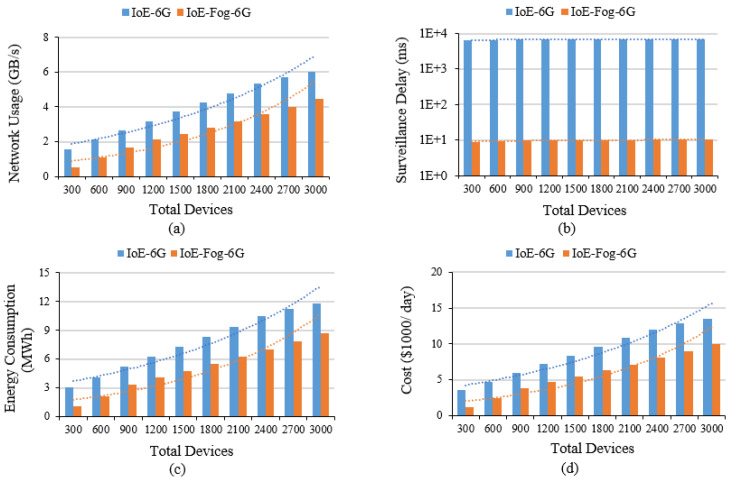
Smart district case study results: (**a**) Total network usage, (**b**) Smart surveillance application average loop end-to-end delay on a log scale, (**c**) Network energy consumption, and (**d**) Estimated energy cost.

This section discusses the Smart District results of both IoE-6G and IoE-Fog-6G scenarios for the 10 configurations simulated in terms of network usage, application loop end-to-end delay, and energy consumption. Figure 8a shows that the difference in network usage between the two scenarios remains study with the growth in the granularity of IoE devices, at an average of 38% reduction in network usage. This clearly shows the role of edge/fog deployment in reducing the pressure on the 6G network, even when the number of IoE devices grows, by performing computation near the users and avoid transferring data to data centers as much as possible.

We evaluated the end-to-end delay of the three applications, Smart Surveillance, Smart Meter, and Smart Bin Figure 8b shows the Smart Surveillance application result, while the other applications results are not presented here as they have a similar pattern which is due to the similarity in the module placement. All applications showed a significant reduction in the end-to-end delay of the application’s main control loop due to the local analysis on the fog. Smart Surveillance delay ranged from 8 to 10 ms for IoE-Fog-6G and from 6 to 7 s for IoE-6G scenario. Smart Meter and Smart Bin delay ranged from 10 to 12 ms for IoE-Fog-6G and from 4 to 5 s for IoE-6G scenario. The slight increase in the delay is due to the growth in the granularity of IoE devices which increases the pressure on fog devices with more workloads arriving at them.

Figure 8c shows the average energy consumption of network data transfer decreased by about 3 MWh for 3000 devices when the fog is deployed in the network at the IoE-Fog-6G scenario. The cost of energy, as shown in Figure 8d, also decreased at the same rate with a total saving of around $1,280,000 per year for 3000 devices.

## 6. Distributed Artificial Intelligence-as-a-Service (DAIaaS)

AI is critical in embedding smartness into smart cities and societies. Due to the exponential increase in the number IoE devices, a pressing need to reduce latencies for real-time sensing and control, privacy constraints, and other challenges, the existing cloud-based AIaaS model, even with fog and edge computing support, is not sustainable. Distributed Artificial Intelligence-as-a-Service (DAIaaS) will facilitate standardization of distributed AI provisioning in smart environments, which in turn will allow developers of applications, networks, systems, etc., to focus on the domain-specific details without worrying about distributed training and inference. Eventually, it will help systemize the mass-production of technologies for smarter environments. We describe in this section DAIaaS and investigate it using four different scenarios.

### 6.1. DAIaaS: Architectural Overview

**Figure 9:**
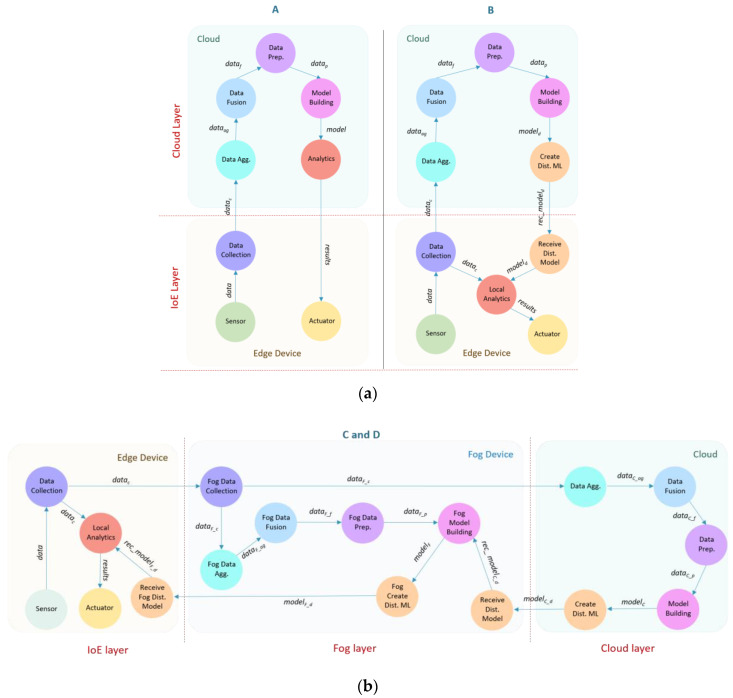
DAIaaS: (**a**) Scenarios A and B and (**b**) Scenarios C and D.

Figure 9 shows four DAIaaS provisioning scenarios (Scenarios A, B, C, and D) for distributed training and inference to investigate various design choices and their performance. DAIaaS comprises several modules that represent typical core operations in AI workflow, including Data Collection, Data Aggregation (Data Agg.), Data Fusion, Data Pre-Processing (Data Prep.), Model Building, and Analytics. In an AI application, data (*data*) generated from various sensors connected to the edge devices are sent to the Data Collection module. The collected data (*data_c_*) is then sent by Data Collection to the Data Aggregation module that will combine them in a unified form and structure. Aggregated data (*data_ag_*) then will be passed to the Data Fusion module where the data from various sources will be fused to reduce uncertainty and produce enhanced forms of the data. Then, fused data (*data_f_*) will be pre-processed by the Data Pre-Processing module where any missing data, noises, and drift are treated, and data are reduced and transformed as necessary. Finally, Preprocessed data (*data_p_*) are passed to the Model Building to train or retrain the model. When the model (*model*) is ready, the Analytics module will represent the inference step where the model is used to produce a decision or prediction as a result (*results*). We discuss next the four scenarios in detail.

### 6.2. Scenario A: Training/Retraining and Inference at Cloud

In the first DAIaaS scenario (Figure 9a), all the data are sent to the datacenter (clouds) to be processed. Therefore, all the AI computation and processing modules are located in the datacenter including Data Agg., Data Fusion, Data Prep., Model Building, and Analytics, except the Data Collection module that will be located on the edges to receive data from sensors. Figure 9a shows these modules, their arrangements in the two network layers, and the workloads between them as a directed graph. Table 9 lists the different workloads passing between the modules and the required resources in terms of the network and CPU computations. We will see in Section 6.5 that this scenario will facilitate a high-level of computing and storage resources, allowing the applications to run higher accuracy models on large volumes of data at the expense of higher delays.

### 6.3. Scenario B: Training/Retraining at Cloud & Inference at Edge

The model is built, trained, and retrained on the cloud in the second DAIaaS scenario (see depiction in Figure 9a, and the workload configuration in Table 9), but a smaller version of the model is built and sent to the edge devices. Edges in this scenario are responsible for inference, and passing the inference results to the actuators. The cloud layer contains the modules: Data Agg., Data Fusion, Data Prep., Model Building, and Create Distributed Model (Create Dist. ML). The Create Dist. ML module is an extra module that generates a smaller version of the model called Dist. model (*model_d_*) that is sent it to the edges. Techniques such as distillation, pruning, and quantization can be used to reduce the model size with minimum effect on model accuracy. The Data Collection, Local Analytics, and Receive Distributed Model (Receive Dist. Model) modules are placed at the edge. The Local Analytics module will use the model received from the cloud to generate results in a real-time manner. The Receive Dist. Model module is required to receive the distributed model (*rec_model_d_*) from the cloud and passed to the Local Analytics.

The Data Collection module will use the Local Analytics module 90% of the time but because the edge-local model will be outdated after a while, it will offload the data to the cloud 10% of the time to retrain the model and receive a new model. We will see in Section 6.5 that this scenario will facilitate a high-level of computing and storage resources, however, the model accuracy will be affected due to smaller, somewhat outdated models running locally on edges with the advantage of faster response times due to analytics at the edge.

### 6.4. Scenarios C and D: Training/Retraining at Cloud and Fog and Inference at Edge

The scenarios C and D contain the extra (fog) layer (see Figure 9b and Table 9). The model building and retraining happens in both cloud and fog layers but the model retraining at the cloud is done less often to reduce latency. The main AI modules (Data Agg., Data Fusion, Data Prep., Model Building, and Create Dist. ML) are located in both the datacenter and the fog. There is no direct communication between the edge and the cloud. The Create Dist. ML module is required in both the cloud and fog layers to create smaller models namely Cloud dist. model (*model_C_d_*) and Fog dist. model (*model_F_d_*). These smaller models are able to fit within the available resources in their parent layers. The Receive Dist. Model modules are needed in the fog to receive the distributed model from the cloud (*rec_model_C_d_*). Similarly, it is needed on the edge to receive the distributed model from the fog (*rec_model_F_d_*). The edges will have the Receive Dist. Model, Data Collection, and Local Analytics modules, which will work similar to Scenario B to generate results in a real-time manner.

Figure 9b shows that both scenarios C and D have the same modules and arrangements, however, they differ in the time they are retrained on the cloud. In Scenarios C and D, the Data Collection module will use the Local Analytics module 90% of the time, as was the case in Scenario B, however, in this case, it will offload the data to the fog layer 10% of the time to retrain the model and receive a new model. In Scenarios C, the fog will retrain the model using the new data received from its edge devices 90% of the time locally and 10% of the time on cloud. In Scenario D, this split is 99% at fog and 1% at cloud. These scenarios provide relatively lower accuracy than Scenarios A and B but with the benefits of lower latencies.

### 6.5. Results and Analysis

**Figure 10:**
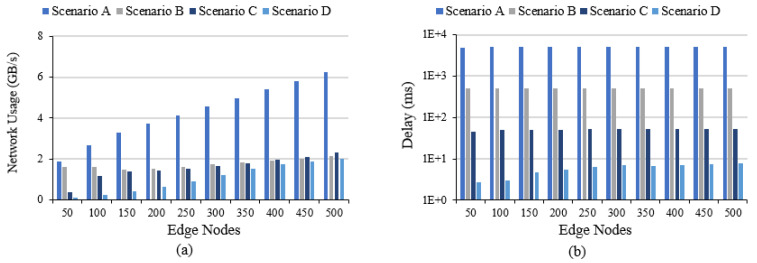
DAIaaS results: (**a**) Total network usage and (**b**) Average loop end-to-end delay for all requests on a log scale.

We now discuss the results for the four scenarios. All four scenarios are investigated using the same number of edge devices (varying between 50, 100, up to 500). Scenarios A and B have no fog devices, while in Scenarios C and D, the number of fogs is fixed at 50. Figure 10a shows the network usage of the DAIaaS model for the four scenarios. Note that the network usage of scenario A is exponentially increasing compared to the other scenarios with the number of edges increases. This clearly shows that offering AI as services on different levels (edge and fog) will reduce the pressure on the 6G network compared to using merely Cloud AIaaS (as in scenario A). In the case of 500 edges, the usage is reduced from 6 GB/s in scenario A to 2 GB/s in scenarios B, C, and D which is a three-fold improvement.

Figure 10b shows the average end-to-end delay for all AI applications in the four scenarios. The number of loops differs in each scenario, and also the frequency of each loop depends on the configuration. Scenario A has one loop, which is the tuple (Data Collection, Data Agg., Data Fusion, Data Prep., Model Building, Analytics, Actuator). Scenario B has two loops one to the cloud for model creation (Data Collection, Data Agg., Data Fusion, Data Prep., Model Building, Create Dist. ML, Receive Dist. Model) and the second in the edge to get the results (Data Collection, Local Analytics, Actuator). Scenario C and D have three loops, one to the cloud for model creation (Fog Data Collection, Data Agg., Data Fusion, Data Prep., Model Building, Create Dist. ML, Receive Dist. Model), the second to the fog, also, for model creation (Data Collection, Fog Data Collection, Fog Data Agg., Fog Data Fusion, Fog Data Prep., Fog Model Building, Fog Create Dist. ML, Receive Fog Dist. Model) and the third in the edge to get the results (Data Collection, Local Analytics, Actuator). Note in Figure 10b that the delay has been reduced significantly from Scenario A (all AI at the cloud) to other scenarios because in the other scenarios data are processed less often in the cloud. Scenario D has the lowest delay of around 8 ms (for 500 edges) as only 1% of the time data travels to the cloud while in scenario A the highest delay of around 5 s (for 500 edges) is because 100% of the time data travels to the cloud.

**Figure 11:**
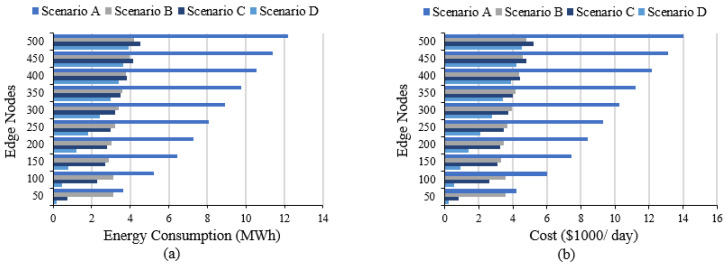
DAIaaS results: (**a**) Network energy consumption and (**b**) Network energy cost.

Figure 11a shows the average energy consumption of the network data transfer for the four scenarios. The energy consumption decreases by more than 7 MWh (for 500 edges) from scenario A at cloud (12 MWh) to scenarios B, C, and D (4 MWh). The cost of energy also decreases at the same rate, as shown in Figure 11b, with a total saving of around $3.1 million per year for 500 edges.

## 7. Conclusions and Future Work

In this paper, we proposed a framework for DAIaaS provisioning for IoE and 6G environments and evaluated it using three case studies comprising eight scenarios, nine applications and delivery models, and 50 distinct sensor and software modules. These case studies have allowed us to investigate various design choices for DAIaaS and helped to identify the performance bottlenecks. The first two case studies modelled real-life physical environments. We were able to see the benefits of various computation placement policies, allowing us to reduce the end-to-end delay, network usage, energy consumption, and annual energy cost by 99.8%, 33%, 3 MW, and 36%, on average, respectively. The third case study investigated various AI delivery models, without regard to the underlying applications. Again, we were able to identify various design choices that allowed us to reduce the end-to-end delay, network usage, energy consumption, and annual energy cost by 99%, 66%, 8 MW, and 66%, on average, respectively. We also showed that certain design choices may lead to lower performance (e.g., higher latencies) at the cost of higher AI accuracies and vice versa.

To the best of our knowledge, this is the first work where distributed AI as a service has been proposed, modeled, and investigated. This work will have a far-reaching impact on developing next-generation digital infrastructure for smarter societies by facilitating standardization of distributed AI provisioning, allowing developers to focus on the domain-specific details without worrying about distributed training and inference, and by helping systemize the mass-production of technologies for smarter environments. The future work will focus on improving the depth and breadth of the DAIaaS framework in terms of the case studies, applications, sensors, and software modules, and AI delivery models, and thereby, on developing new strategies, models, and paradigms for the provision of distributed AI services.

## Figures and Tables

**Table 1:** Summary of relevant research.

Research	IoE	Edge/Fog	Smart Societies	6G	Distributed AI (DAI)	as a Service (aaS)	AIaaS	DAIaaS
Letaief et al. [13]		x		x	x	x		
Smith and Hollinger [40]		x	x		x			
AlSuwaidan [49]	x		x			x		
Lv and Kumar [50]	x	x		x				
Aiello et al. [51]	x		x			x		
Nath et al. [61]	x	x	x		x			
Wang et al. [62]	x	x	x		x			
Badii at al. [63]	x	x	x					
Ahad et al. [77]	x		x					
Casati et al. [42]			x			x	x	
Milton et al. [43]	x		x			x	x	
This work	x	x	x	x	x	x	x	x

**Table 2:** Device configurations.

Device Parameter	Cloud (VM)	Fog Device	Edge Device
MIPS	220,000	50,000	5000
RAM (MB)	40,000	4000	1000
Uplink Bandwidth (Mbps)	100	10,000	10,000
Downlink Bandwidth (Mbps)	10,000	10,000	10,000
Busy Power (W)	16 × 103	107.339	87.53
Idle Power (W)	16 × 83.25	83.4333	82.44

**Table 3:** Smart surveillance: workload configuration.

Workload Type	Source Module	Destination Module	CPU Requirement (w_c_) (MI)	Network Requirement (w_n_) (Bytes)
*vid_strm*	Camera	Motion Detector	1000	20K
*motion_vid_strm*	Motion Detector	Obj Detector	2000	2000
*detected_obj*	Obj Detector	User Interface	500	2000
*obj_location*	Obj Detector	Obj Tracker	1000	100
*cam_ctrl*	Obj Tracker	Camera Ctrl	50	100

**Table 4:** Links latency configurations.

Link (*L*)	Latency (ms)
Cloud-Gateway	100
Gateway-Fog	2
Fog-Edge	2
Edge-Sensor/actuator	1

**Table 5:** Estimated network energy consumption [87].

Estimated Energy Consumption	2010 (kWh/GB)	2020 (kWh/GB)	2030 (kWh/GB)
Best	5.65	0.05	0.002
Worst	14.78	1.04	0.048
Average	10.22	0.54	0.025

**Table 6:** Smart Airport: Sensors Configuration.

	Camera	Barcode Reader	Counter Device
**Workload type**	*vid_strm*	*barcode*	*info*
**Distribution (ms)**	Deterministic Distribution (5)	Uniform Distribution (5,20)	Uniform Distribution (5,20)

**Table 7:** Smart airport: workloads configuration.

Workload Type	Source Module	Destination Module	CPU Req. (MI)	Network Req. (Byte)
*barcode*	Barcode Reader	Boarding Processor	100	1000
*passenger_info*	Boarding Processor	Authenticator (Auth.)	2000	1000
*gate_ctrl*	Authenticator	Gate Ctrl	100	100
*auth_info*	Auth. Info Provider	Authenticator	100	100
*info*	Counter Device	Check Info	100	1000
*passenger*	Check Info	Passenger Processing	500	1000
*passenger_info_req*	Passenger Processing	Auth. Info Provider	1000	1000
*passenger_info_res*	Auth. Info Provider	Passenger Processing	1000	100
*counter control*	Passenger Processing	Counter Ctrl	100	500

**Table 8:** Smart District: Workloads Configuration.

Workload Type	Source Module	Destination Module	CPU Req. (MI)	Network Req. (Byte)
*meter_ reading*	Meter	Meter Monitor	100	500
*outage_ status*	Meter Monitor	Outage Notifier	500	2000
*meter_ status*	Meter Monitor	Elect Controller	1000	2000
*elect_analysis*	Elect Controller	User Interface	1000	500
*ctrl_params*	Elect Controller	Meter Ctrl	500	50
*bin_ reading*	Bin	Bin Monitor	100	500
*full_status*	Bin Monitor	Full Notifier	200	2000
*bin_ status*	Bin Monitor	Bins Coord	800	2000
*waste_cond*	Bins Coord	User Interface	1000	500
*ctrl_params*	Bins Coord	Bin Ctrl	500	50

**Table 9:** DAIaaS: Workloads configuration.

Workload Type	Source Module	Destination Module	CPU Req. (MI)	Network Req. (Byte)
Scenario A				
*data*	Sensor	Data Collection	100	RD = 20 K
Collected data (*data_c_*)	Data Collect	Data Aggregation	200	RD
Aggregated data (*data_ag_*)	Data Aggregation	Data Fusion	100 K	DA = RD × E
Fused data (*data_f_*)	Data Fusion	Data Prep.	150 K	DF = DA × 0.80
Preprocessed data (*data_p_*)	Data Prep.	Model Build	150 K	DP = DF × 0.50
*model*	Model Build	Analytics	200 K	1 MG
*results*	Analytics	Actuator	100 K	1000
Scenario B				
*data*	Sensor	Data Collection	100	RD = 20 K
Collected data (*data_c_*)	Data Collection	Data Aggregation	200	RD
Aggregated data (*data_ag_*)	Data Aggregation	Data Fusion	100 K	DA = RD × E
Fused data (*data_f_*)	Data Fusion	Data Pre-Processing	150 K	DF = DA × 0.80
Preprocessed data (*data_p_*)	Data Pre-Processing	Model Building	150 K	DP = DF × 0.50
*model*	Model Building	Create Dist. ML	200 K	M = 1 MB
Dist. model (*model_d_*)	Create Dist. ML	Receive Dist. Model	200 K	DM = M/E 5K < DM < 50K
Rec dist. model (*rec_model_d_*)	Receive Dist. Model	Local Analytics	1000	DM
Collected data (*data_c_*)	Data Collection	Local Analytics	200	RD
*Results*	Local Analytics	Actuator	3000	1000
Scenarios C and D				
*data*	Sensor	Data Collection	100	RD = 20000
Cloud collected data (*data _C_c_*)	Fog Data Collection	Data Aggregation	200	FDC = RD × E
Cloud aggregated data (*data _C_ag_*)	Data Aggregation	Data Fusion	100 K	DA = FDC × F
Cloud fused data (*data _C_f_*)	Data Fusion	Data Pre-Processing	150 K	DF = DA × 0.80
Cloud preprocessed data (*data _C_p_*)	Data Pre-Processing	Model Building	150 K	DP = DF × 0.50
Cloud model (*model_C_*)	Model Building	Create Dist. ML	200 K	M = 1 MB
Cloud dist. model (*model_C_d_*)	Create Dist. ML	Rec. Dist. Model	200 K	DM = M/F 20K < DM < 200 K
Rec cloud model (*rec_model_C_d_*)	Rec. Dist. Model	Fog Model Building	10 K	DM
Collected data (*data_c_*)	Data Collection	Fog Data Collection	200	RD
Fog collected data (*data_F_c_*)	Fog Data Collection	Fog Data Aggregation	200	FDC = RD × E
Fog aggregated data (*data _F_ag_*)	Fog Data Aggregation	Fog Data Fusion	20 K	FDA = FDC × E
Fog fused data (*data _F_f_*)	Fog Data Fusion	Fog Data Pre-Processing	30 K	FDF = FDA × 0.80
Fog preprocessed data (*data _F_p_*)	Fog Data Pre-Processing	Fog Model Building	30 K	FDP = FDF × 0.50
Fog model (*model_F_*)	Fog Model Building	Fog Create Dist. ML	40 K	FM = M/F 20 K < FM< 200 K
Fog dist. model (*model_F_d_*)	Fog Create Dist. ML	Rec. Fog Dist. Model	40 K	DFM = FM/E 5 K < DFM < 50 K
Rec fog model(*rec_model_F_d_*)	Rec. Fog Dist. Model	Local Analytics	1000	DFM
Collected data (*data_c_*)	Data Collection	Local Analytics	200	RD
*results*	Local Analytics	Actuator	3000	1000
